# Principles in Silicate Work

**Published:** 1911-05-15

**Authors:** C. M. Baldwin

**Affiliations:** Chicago, Ill.


### PRINCIPLES IN SILICATE WORK.

BY C. M. BALDWIN, D.D.S., CHICAGO, ILL.

In a new field of work, materials and technique frequently and sometimes radically change in a short time, and silicate work has been no exception.

It is comparatively easy to look back and see that the inexperience of the entire profession necessarily made it impossible to correctly determine relative values of the various silicate cements, or at once to established best methods of operating.

We may not realize as yet the full value of the best silicate fillings, but we know that they may be in pratically perfect condition after more than five years service.

It is just as necessary for the dentist to intelligently choose materials and know that they are in good condition when used as it is for him to develop skill in using them.

### ORIGINAL METHOD DANGEROUS.

A fair trial of this method soon revealed its weakness and dangers, as the almost universal experience was discoloration of fillings, defective margins and chalky or granular surfaces.

These fillings usually appeared beautiful and entirely satisfactory when finished, making it rather difficult to know causes of failures seen a few months later.

### REQUIREMENTS FOR SUCCESSFUL WORK.

The greatest need at present is not to have materials improved, but that we have a better understanding of the work, so as to enable each operator to bring average work up reasonably near the best results.

The most successful operators today agree upon requirements and principles involved for producing best work.

### PRINCIPLES OF WORK.

The great fundamental principle controlling successful work is, that from purchase of materials to completion of operation, the characteristic qualities of materials be preserved practically uninjured.

All operators must be limited by nature of materials used, but all do not give equally good attention to keeping them in perfect condition. Intelligent care of materials is one of the most necessary features for successful work, as skillful use cannot overcome failures caused by damaged materials.

### LIQUID.

The clear liquid, free from cloudiness or precipitate when purchased, removes one of the early handicaps. Some operators used original liquid after too heavy precipitate had formed, which prevented normal chemical action. Properly protected the liquid may be used to about its last fifth, without cloudiness or crystals forming.

These changes will occur when liquid is needlessly exposed to air, or left around mouth of bottle, it transfers effect of air to liquid within, as upon opening it re-enters bottle.

With materials properly protected, the principles controlling the mix, and stickiness of the operation, are of the greatest importance.

### PRINCIPLES OF MIXING.

The best mix should develop all the best qualities of the materials and allow the operator to use it skilfully. Three distinct features control the mix.

Moistening powder with the acid begins and completes mix, when all powder particles added are included.

Time consumed and how distributed throughout mix, influences setting.

Air exposure on slab considerably hastens crystalization.

As we understand and apply these principles will we adjust proportions of powder to liquid, affect setting process, density of fillings and value generally. Have separate agate or glass slab for this work, and keep it free from old materials from previous mixes, soot, dust, lint, etc. Use bone or ivory spatula one-quarter inch wide or less, with dull knife edges and slightly pointed. Larger spatulas for this mix are proportionately disadvantageous.

Always place slab in good light. Increasing size of mix retards setting. A decided advantage is gained by using two drops of liquid, or more, if needed, for exceptionally large fillings. Bring powder into liquid as continuously and quickly as it can be moistened. Avoid grinding or spreading mass and loss of time all through process. This manner of mixing permits considerable more powder being used than was possible when so much time was used to spread and grind, causing mass to set before operation was started. Last powder used should be in small quantities, and always leave mass more moistened than when ready for placing in cavity. In extremely hot weather and excessively dry atmospheric conditions mass should show slight free acid appearance after all powder has been added, but before final mix. If mass is spread out at this time more or less dry particles of powder will be seen. To thoroughly moisten these requires a final kneading for about thirty seconds, and a sufficiently moist mass.

Moistening dissolves more or less of the powder, or is the reverse of crystalization. Avoiding loss of time and spreading mass to thin layer exposed to air minimizes setting, so that the completed mix should be very sticky.

Finally, to prevent unnecessary air exposure, gather mass into cube form without any projecting thin edges, having it stand nearly as high as it is broad on slab. A suitable slice should be cut from one side and packed, and each succeeding piece should be removed from the freshly exposed surface. The opposite side exposed to air since cube was formed will be decidedly crust-like compared with inside remainder of mass, which has not been exposed. The eperator should never use the crust portion of mass and ought to mix large enough mass to avoid it. Make a second mix rather than use one that is faulty.

### TANTALUM INSTRUMENTS.

The new tantalum instruments provide the much needed, strong, thin blades and small points. A few of these, together with some tortoise shell point instruments, where greater bulk is unobjectionable, makes steel instruments entirely unnecessary.

### DRYNESS.

An absolutely indispensable principle for successful work, demands perfect dryness of filling until crystalization has sufficiently developed to protect it.

### STICKINESS.

It should be thoroughly understood that the peculiar stickiness of this material was the only reason for introducing the preventive method of operating, viz., vaseline on strips and instruments to prevent adhesion.

Stickiness, then, is the new operative feature and real handicap that must be overcome, to which was added the exclusion of steel instruments to avoid discoloration.

Polished steel instruments, carefully used, have caused much less discoloration than the original oil strip method.

### STICKINESS CONTROLLED

Clean instruments necessarily drag filling when in contact with sticky surface while building piece upon piece.

Nothing whatever of any kind is needed or should be put on instruments to prevent adhesion. Just as surgeons use artery forceps and sponges to control their operative field, must we control stickiness, our handicap in operating.

### DETAILS OF PACKING.

With a small blade, suitable size pieces are cut from cube mass on slab, placed in cavity and securely fixed by strong adhesion. Meanwhile the packing instruments untouched by sticky material, remain on the table.

While en route from mouth to table, with cambric (no starch) or Red Cross napkin, quickly remove soft, sticky coating from point, thus prepairing it for repeated use. Each instrument having any material adhere to it is similarly wiped before exchange of instruments. The wiping, or short dragging stroke, used for all interior packing and building filling, should be with side of instrument on surface of material, and attach marginal portions first. As much as possible it should be a pull towards handle rather than a push towards end of instrument. The pressure is always towards walls or margins and the shorter the stroke the better, lifting instrument from surface while moving.

Surface packing requires large surface instruments to prevent adhesion and quickly place crystals closely and firmly together. Quickly touching and removing instruments from a rather gummy surface, packs instead of raises surface, provided instrument is not allowed to slip. All wiping, slipping strokes should be carefully avoided, as they drag and cause separation of crystals when too late to perfectly readjust them, causing granular surfaces and defective margins which later become stained.

Begin surface packing at margins, reinforcing adhesion, and density is increased, both at margins and over entire surface. It is desirable to repack or continue packing surface for short time after it has been given fine grain appearance, using judicious pressure to avoid disturbing crystalization. The carrying instrument should remove more or less excess while placing each piece, but all material packed should remain part of filling until hard.

### STICKINESS VS. DENSITY.

Instead of density being lessened by increased stickiness of mix the reverse will be found nearer correct. Density and fine grained surface will be improved by increased powder to liquid, and by forming granules into a thoroughly compact mass before gumminess disappears from surface, and maintaining moderate pressure throughout early setting period. Advanced chemical action, while packing, makes heavier pressure necessary and should be avoided.

### FINISHING OR GRINDING PROCESS.

Never disturb crystalization when finishing filling. About fifteen minutes after packing, filling should be sufficiently set so that any necessary use of disks, strips or stones will not injure it. Until grinding begins fillings remains without coating of any kind, but all disks, strips and stones are lubricated with vaseline, paraffine, cocoa butter or soapstone powder. The dry method increases danger of chipping margins. Carborundum, too coarse grits, stones that pound or have been used on metals will injure fillings. Final polish requires cuttlefish (00), Arkansas stones and celluloid disks for all labial surfaces.

To protect from saliva when rubber is removed, leave same kind of oil on filling that was used when polishing.

Grinding removes hardest section of filling and the polished surface ought to be allowed fifteen or more minutes before removal of rubber.

Thirty minutes protection by rubber and postponing finishing to future sitting requires but very few minutes of operator's time at first sitting in addition to cavity preparation. This gives uninterrupted half hour when operator requires it for other work, affords safety to filling and especially should be adopted when using as best form of temporary work.

Every extra five minutes beyond minimum protection will add to service of filling.

Alcohol, water, saliva, etc., injure filling proportionately as they are in contact with it during setting process.

### DISCOLORATIONS.

There are three classes of discolorations:

*First*—Foreign bodies (dirt, soot, metals, etc.) entering materials while in bottles, on slab, packing or finishing fillings, positively discolor.

*Second*—Chalky, rough or granular surfaces become stained with food, tobacco and medicines.

*Third*—Marginal stains may include the other two classes, but leaky fillings or detached margins are the chief causes.

### PLAN OF WORK.

1. Patient who appreciates beauty of work.
2. Diagnosis favorable to friable material and protection by rubber.
3. Cavity—general retentive form, with square margins—never beveled.
4. Materials kept in good condition.
5. Instruments—non-metallic or tantalum—suitable sizes and forms.
6. Slab—agate or glass—clean.
7. Spatula—bone $\frac{1}{4}$ inch wide or less.
8. General cleanliness—preparation entire equipment for use.
9. Operative conditions—1, rubber protection; 2, cavity thoroughly dry; 3, equipment conveniently placed before mixing.

10. Mix—1, materials on slab; 2, bottles closed; 3, mix (a) moisten powder, (b) knead mass, (c) gather into cube form.
11. Packing—1, carrying instrument removes piece from cube; 2, places in cavity; 3, wiped with cambric; 4, packing instruments used and wiped alternately with carrying instrument; 5, surface packing.
12. Crystalization—about fifteen minutes.
13. Grinding—1, sandpaper and cuttle fish disks and strips; 2, corundum, gem, Arkansas stones; 3, celluloid disk—final polish; 4, all lubricated with (a) vaseline, (b) paraffine, (c) soapstone powder.
14. Crystalization—1, minimum fifteen minutes—longer time desirable; 2, thirty minutes in one period when finished next sitting.
15. Protect filling—1, same kind of oil used when grinding; 2, oily coating or good varnish when finish is postponed.

### MISCELLANEOUS CONSIDERATIONS.

The high standard of modern dentistry has been achieved only after many years of development.

The individual's success depends upon ability to understand and apply the knowledge gained. Years of college instruction followed by years of experience usually are necessary to train anyone to become a skillful operator, with materials that have been used, studied and improved by successive generations.

### HASTY CONCLUSIONS.

In contrast to such careful preparation for work, many users of silicate cements have taken up this new field of work very much as though it was a "grab bag affair" or get "something for nothing" proposition. Usually inexperience must pay its price, while experience carries a premium, valuable according to who possesses it.

It is a rather common experience to hear dentists' opinions based upon their own and other's failures, frequently without knowing which kind had been used, or considering method of insertion or details of operation.

One may classify old fillings, but to determine from appearances only, just which particular kind in its class had been used, is practically impossible.

### FAILURES.

As a rule radical failures of gold and amalgam fillings are blamed against the operator.

During the first few years failures in this new work quite generally were considered due to weakness of materials.

The added experience of recent years indicate most conclusively that the vast majority of radical failures are caused by the operator. The most successful operators have comparatively very few such failures, and consider most of them as avoidable.

Old habits largely control the skillful dentist when attempting new kinds of work.

Necessarily more or less failures follow until he knows the nature of the new materials, and adapts himself to the new conditions. The original method was exceedingly simple, while recently some writers have exaggerated the difficulties, both chemically and operative. Undoubtedly midway between these extreme views is a better appreciation of what is required to be successful in this work.

Those operators who promised "better than gold" without qulifications or other considerations, have had their disappointments when results were unsatisfactory.

The first mistake was poor salesmanship, and the second, they did not deliver the goods as represented. Disappointments are only the difference between expectations and actual results.

### PERMANENCY VS. ESTHETICS.

For many years in this country we have had considered permanency as the test of successful work, but continued use of porcelain crowns, inlay and silicate cements has developed an increasing appreciation and demand for esthetic work.

When permanency is the main consideration, there can be no question or doubt but that metal fillings or inlays should be used. When esthetic features control, or there are reasonable objections to alternate operations, should be the deciding factors for selecting this filling material.

The demand for esthetic work is just as reasonable as for the so-called permanent work.

Permanency, however, should always be a very close second requirement, unless the material is intentionally used for temporary purposes.

Because these fillings are in good condition after five or more years' service, should be sufficient incentive to very earnestly endeavor at least to dup.icate such results and make us less satisfied when fillings fail in less time. When fillings fail, try to find the cause, so as to eliminate it from future work.

Corduroy, duck, homespun clothes and cowhide boots may be necessary to resist rough usage, but there are many people who prefer broadcloth, silks, satins and laces and feel well pleased with the kind of service they give. We should not confuse conditions or requirements.

Misrepresentations intentionally or otherwise cause dissatisfaction, while a perfect understanding avoids it.

### ATMOSPHERIC INFLUENCES.

The mass on slab is considerably influenced by varying atmospheric conditions.

The degree of temperature in office, whether natural or artificial, is almost the sole cause. Higher temperatures proportionately hasten setting, while lower degrees retard

Dryness and heat of the tooth also accelerates chemical action. Use every safe means to retard setting while mass is on slab, especially in extremely hot weather. Early morning appointments would give operative advantage.

Remove bottles from sun exposed cabinet. An hour before using place slab and closed bottles on a cold surface, viz.: mosaic floor, marble, or heavy glass top table. Excessively chilled slab might endanger mix in humid atmosphere. Never allow strong current of air from open window or electric fan to pass over slab while mixing. The mix as described retards setting and uses such a large proportion of powder that less may be used when necessary to give sufficient operative time and proper consistency of mass. First, however, adopt all other suitable means.

### MARGINS.

Real stickiness is needed to seal margins, avoid chipping while finishing, and is the protection against leaky or discolored margins. Do not detach with later packing or finishing.

It was a very general result with the original method. However, today some very successful operators use the dry celluloid strip method, holding carefully until setting releases it, afterward grinding excess as usual.

### NEW MATERIALS.

Frequently new materials have been known to give much better results than the same operator could produce one year later with materials from his cabinet. Old materials, or new liquid with old powder, or vice versa, too frequently mean poor results.

Experience has taught the necessity of removing base metal of die or counter die before soldering gold. It is just as necessary to prevent contamination of these new materials and protect them from weakening influences.

Try opening new bottles of powder and liquid for same mix, and notice improvement. Close bottles, using pressure and twist before returning to cabinet. Improve next month's filling by caring for materials today. Even in commercial lines, many articles formerly sold in bulk are protected by enclosure in sealed packages. One may have satisfactory service carrying a watch without being a jeweler, and it is just as unnecessary to be a chemist to give these materials the simple protection required.

Early enthusiasm led some to insert the silicates in a very large proportion of all cavities filled, before they had an opportunity to observe results, consequently failures soon discouraged such a practice.

Confidence in porcelain inlay work, while considerably decreased, continues such as it is because with all its difficulties and failures, the operator is using old familiar materials and realizes that operative features need correcting if results are to improve. A feature not to be lightly overlooked, is that physical and nervous strain for both patient and operator is reduced to the minimum by this class of work.

### A FEW DETAILS.

Secure ample space for simple proximal cavities. When space gained is less than required with size of instruments to be used, cut piece from gelatine medicine capsule, turn over proximal and labial surfaces of tooth opposite cavity. It saves all space needed for finishing, which is postponed to another sitting. Leave gelatine piece in position when removing rubber, as saliva soon dissolves it.

Never use steel instruments to cut away excess of filling within margins. Clean chisels may be used to chip away thin brittle excess beyond margins. Push instrument through excess and away from margins, but never toward them.

The last chance to protect liquid is when on slab before mixing. Carefully observe glistening surface and remove any small foreign body, viz.: dust, lint, soot or paraffine.

Coat old fillings with vaseline, paraffine or varnishes as indicated, to protect from medicines, or dryness under the rubber.

Veneers mechanically retained in old gold fillings (needing repair), and inlays, combine beauty and strength. Never attempt to fill a comparatively large cavity in contrast with a mall opening, just because using a plastic material.

Before placing silicate fillings allow volatile drugs in root fillings and medicated cements to evaporate, then cover with quick setting oxyphosphate.

The free acid causes effervesence on pearl and some forms of onyx, indicating they should not be used as instruments, spatulas or slabs. *Dental Review.*

